# Knock Out of CGN and CGNL1 in MDCK Cells Affects Claudin-2 but Has a Minor Impact on Tight Junction Barrier Function

**DOI:** 10.3390/cells12152004

**Published:** 2023-08-05

**Authors:** Marine Maupérin, Ali Sassi, Isabelle Méan, Eric Feraille, Sandra Citi

**Affiliations:** 1 Department of Molecular and Cellular Biology, Faculty of Sciences, University of Geneva, 1205 Geneva, Switzerland; 2 Department of Cellular and Metabolic Physiology, Faculty of Medicine, University of Geneva, 1205 Geneva, Switzerland

**Keywords:** cingulin, paracingulin, permeability, sodium, claudin-2

## Abstract

Cingulin (CGN) and paracingulin (CGNL1) are cytoplasmic proteins of tight junctions (TJs), where they play a role in tethering ZO-1 to the actomyosin and microtubule cytoskeletons. The role of CGN and CGNL1 in the barrier function of epithelia is not completely understood. Here, we analyzed the effect of the knock out (KO) of either CGN or CGNL1 or both on the paracellular permeability of monolayers of kidney epithelial (MDCK) cells. KO cells displayed a modest but significant increase in the transepithelial resistance (TER) of monolayers both in the steady state and during junction assembly by the calcium switch, whereas the permeability of the monolayers to 3 kDa dextran was not affected. The permeability to sodium was slightly but significantly decreased in KO cells. This phenotype correlated with slightly increased mRNA levels of claudin-2, slightly decreased protein levels of claudin-2, and reduced junctional accumulation of claudin-2, which was rescued by CGN or CGNL1 but not by ZO-1 overexpression. These results confirm previous observations indicating that CGN and CGNL1 are dispensable for the barrier function of epithelia and suggest that the increase in the TER in clonal lines of MDCK cells KO for CGN, CGNL1, or both is due to reduced protein expression and junctional accumulation of the sodium pore-forming claudin, claudin-2.

## 1. Introduction

Tight junctions (TJs) form a continuous intercellular circumferential belt in the apical region of epithelial cells and consist of transmembrane proteins, a cytoplasmic plaque composed of scaffolding and adaptor proteins, and cytoskeletal filaments anchored to cytoplasmic adaptors [1,2,3]. The canonical function of TJs is to form a semipermeable barrier between apical and basolateral extracellular compartments, whereas non-canonical functions of TJs comprise the regulation of signaling and activities of Rho family GTPases [4,5]. The TJ barrier is formed by proteins of the claudin family, which polymerize into TJ strands and regulate the passage of ions, water, solutes, and cells across epithelial tissues [3,6,7]. It is controlled by the contractility of the adherens-junction-associated actomyosin cytoskeleton, is altered in disease, and comprises “pore” and “leak” pathways [8,9]. The pore pathway allows the size- and charge-selective passage of ions and solutes through channels made of specific claudin isoforms, which are differentially expressed in tissues and tissue sub-compartments [10]. For example, claudin-2, a channel-forming claudin, promotes the permeability to cations and water in the proximal tubule of the kidney [11].

Claudins are polymerized at a TJ on a scaffold of ZO proteins, which in turn interact with different junctional adaptors and signaling proteins, including cingulin (CGN) and paracingulin (CGNL1, JACOP) [12,13,14,15,16]. Although the role of claudins in TJ barrier’s function has been extensively characterized, less is known about the role of adaptor proteins in their direct or indirect functional interactions with claudins, with the actomyosin cytoskeleton and with different signaling pathways. For example, CGN has been implicated in the regulation of the endothelial barrier function through its ability to sequester GEF-H1 at TJs to suppress RhoA activity, resulting in increased stress-induced barrier disruption in CGN-depleted cells [17,18,19]. However, the role of CGN and CGNL1 in the barrier function of epithelial cells and tissues is less well understood. Analysis of transepithelial electrical resistance (TER) shows that knock down (KD) of CGN in MDCK cells has no detectable effect on the steady-state barrier function of TJs [20], whereas cells depleted of CGNL1 show a change in the dynamics of barrier formation, but no change in TER in the steady state [21]. In CGN-knock out (KO) embryoid bodies and mice, no changes were detected in the epithelial tissue architecture and TJ barrier function, as assessed by flux of fluorescent tracers, measurement of electrical resistance of mucosal preparations, and serum endotoxemia [22,23]. To better understand the role of CGN and CGNL1 in TJ barrier function, we analyzed the barrier function properties of KO MDCK clonal cell lines for either CGN, CGNL1, or both. Our results show a small increase in TER in KO clonal lines, a decreased Na^+^ flux and claudin-2 protein expression and accumulation at TJs, and no change in the permeability of larger solutes.

## 2. Materials and Methods

### 2.1. Cell Culture

MDCK (Madin-Darby Canine Kidney type II, female) cells were cultured in Dulbecco’s Modified Eagle’s (DMEM) medium supplemented with 10% Fetal Bovine Serum (FBS), 1% non-essential amino acids (NEAA), 100 units/mL penicillin, and 100 µg/mL streptomycin (P/S) at 37 °C under 5% of CO_2_ [24]. Every 2 days, the medium was replaced with fresh medium. KO clonal cell lines for either cingulin (CGN), paracingulin (CGNL1), or both were generated using the CRISPR/Cas9 gene editing method as previously described [25]. The WT control clonal lines were selected using the same procedure as for the KO lines and confirmed as WT by immunoblot and immunofluorescence analyses. Two clonal cell lines were used per conditions: CGN-KO clone 1 and 2 (clone #1G8, #1H9, respectively); CGNL1-KO clone 1 and 2 (clone #2-2, #2-5, respectively); and double-KO clone 1 and 2 (clone #11C9, #21D3, respectively). The data for the 2 WT clonal lines were pooled together for TER, dextran flux, and dilution potential analyses.

### 2.2. Measurement of Transepithelial Resistance (TER) and Paracellular Flux Assay

To measure the TER of MDCK monolayers in the steady state, cells were plated in triplicate in 6.5 mm Transwell filters (Corning Costar, Sigma-Aldrich Chemie. Buchs, Switzerland, #3450) at a density of 2 × 10^4^ cells/well. On day 7, TER was measured using a Millicell-ERS probe (Merck Millipore, Schaffausen, Switzerland) and a Millicell-ERS Volt-Ohm meter (Millipore, Burlington, MA, USA).

To measure the paracellular flux of 3 kDa fluorescein-dextran, cells were plated in triplicate in 12 mm Transwell filters (Corning Costar, Sigma-Aldrich Chemie, Buchs, Switzerland, #3460) at a density of 7 × 10^4^ cells/well. On day 7, the TER was measured to verify cell confluency. Cells were then washed once with warm Hanks buffer (Gibco, Thermofisher Scientific, Geneva, Switzerland, #14025-050) and incubated for 30 min at 37 °C with new warm Hanks buffer. Then, cells were incubated at 37 °C with a solution of 1 mg/mL 3 kDa fluorescein-dextran (Invitrogen, Thermofisher Scientific, Geneva, Switzerland, #D3305) and placed in the apical compartment, while the basal compartment contained Hanks buffer. At 1 h, 2 h, and 3 h, 100 µL of solution in the basal compartment was collected and replaced with Hanks buffer. The fluorescence of the samples was measured (excitation: 494 nm, emission: 521 nm) using the Cytation3 reader. The 3 kDa fluorescein-dextran permeability was calculated using the following formula [26]:

Perm_app_ = ((final 3 kDa fluorescein-dextran in basal compartment (ng/mL)) × 10^6^)/(filter area (1.12 cm^2^) × (initial 3 kDa fluorescein-dextran in apical compartment (ng/mL)) × time (s)) (1)

To measure the TER during the calcium switch, cells were plated in triplicate in 6.5 mm Transwell filters at a density of 2 × 10^4^ cells/well. On day 7, cells were washed 3 times with Ca^2+^-free DPBS (Gibco, Thermofisher Scientific, Geneva, Switzerland, #14190-094) and incubated for 20 h in Ca^2+^-free medium S-MEM (Gibco, Thermofisher Scientific, Geneva, Switzerland, #11380-037), 10 mM Hepes, 5% dialyzed FBS, 2 mM L-glutamine, and 2 µM EGTA, 1X NEAA, 1X P/S. After 20 h, corresponding to T = 0, the TER was measured and the Ca^2+^-free medium was replaced by normal medium (calcium switch). The TER was then measured at T = 0.5 hr, 1 h, 2 h, 3 h, 4 h, 6 h, 8 h, 24 h, 48 h, 32 h, and 48 h after the calcium switch [27].

To measure the TER during the calcium depletion protocol, cells were plated in triplicate in 6.5 mm Transwell filters at a density of 2 × 10^4^ cells/well. On day 7, the TER was measured (T = 0). Then, cells were washed 3 times with Ca^2+^-free PBS and transferred into Ca^2+^-free medium (calcium depletion). The TER was measured at T = 5 min, 15 min, 30 min, 60 min, and 120 min after the calcium depletion.

### 2.3. Dilution Potential of NaCl

To measure transepithelial potential difference of MDCK monolayers, cells were plated in 12 mm Snapwell polyester filters (Corning Costar, Sigma-Aldrich Chemie, Buchs, Switzerland, #3801) at a density of 7 × 10^4^ cells/well. On day 7, Snapwell rings were detached and mounted in Ussing chambers (Physiologic Instruments, Reno, NV, USA, #P2300) connected to a VCC MC6 multichannel voltage/current clamp through silver/silver chloride electrodes and 3 M KCl agar bridges. The transepithelial potential was recorded by a Quick Data Acquisition DI100 USB board (Physiologic Instrument, Reno, NV, USA,). Cells in Snapwell were equilibrated for 1 h in buffer A (120 mM NaCl, 10 mM NaHCO_3_, 5 mM KCl, 1.2 mM CaCl_2_, 1 mM MgCl_2_, 10 mM Hepes, pH = 7.4). To inhibit the transcellular ion flux, 100 µM amiloride (Sigma-Aldrich Chemie, Buchs, Switzerland, #A-7410) and 100 µM 4,4′-diisothiocyanatostilbene-2,2′-disulfuronic acid (DIDS) (Sigma-Aldrich Chemie, Buchs, Switzerland, #D3514) were added into the apical compartment 30 min before starting diffusion potential measurements. Under a current clamp at 0 µA, half of the buffer A in the basal compartment was diluted by buffer B (240 mM mannitol, 10 mM NaHCO_3_, 5 mM KCl, 1.2 mM CaCl_2_, 1 mM MgCl_2_, 10 mM Hepes, pH = 7.4) from 130 mM to 65 mM NaCl, and the diffusion potentials were measured. The solutions were constantly bubbled with 95% O_2_ and 5% CO_2_ and maintained at 37 °C [28]. The relative permeability ratio for Na^+^ over Cl^−^ (P_Na+_/P_Cl_^−^) and the absolute permeabilities of Na^+^ and Cl^−^ were calculated as described in [29] using the transepithelial resistance measured during the same experiment.

### 2.4. RNASeq Analysis

For RNA isolation, 3 × 10^5^ cells were cultured in each well of 6-well plates. After 3 days, cells were washed with PBS, trypsinized, and pelleted at 5000 g for 5 min. The supernatant was discarded, and RNA was extracted using a NucleoSpin^®^ RNA kit (Macherey-Nagel, Oensingen, Switzerland, #740955.50). A Qubit fluorimeter (Thermofisher Scientific, Geneva, Switzerland) was used for RNA quantification, and a Bioanalyzer (Agilent Technologies, Basel, Switzerland) was used to measure RNA integrity. A library was prepared with 500 ng of total RNA and the TruSeq mRNA stranded kit (Illumina, San Diego, CA, USA). The library molarity and quality were measured using Qubit and Tapestation (DNA high sensitivity chip). Library sequencing was performed using a Hiseq 4000 Illumina sequencer calibrated with an average of 25 × 10^6^ SR100 reads per sample. Differential gene expression mapping and quantification were performed by aligning the reads to the NCBI Canis Lupus Familiaris canFam3 genome (NC_006583.3) with STAR v.2.7.0. The gene expression quantification was obtained with HTSeq v.0.9.1. The differential expression was analyzed using the R/Bioconductor edgeR package. The counts were normalized according to the library size and filtered. Genes with a count of above 1 count per million reads in at least 3 samples were selected for analysis. The differentially expressed genes tests were performed with a general linear model (GLM) using a negative binomial distribution. Differential gene expression was conferred when the fold change (FC) was 2-fold, with a 5% false discovery rate (FDR) in Benjamini–Hochberg multiple testing correction [24].

### 2.5. Immunoblot Analysis

Confluent cells in 10 cm dishes were lysed for 10 min at 4 °C with 500 µL of RIPA buffer (150 mM NaCl, 40 mM Tris-HCl, 1% Triton X-100, 10% glycerol, 2 mM EDTA, 0.2% SDS, 0.5% deoxycholate, pH = 7.5) supplemented with fresh protease inhibitor cocktail (Thermofisher Scientific, Geneva, Switzerland, #A32965). Total lysates were sonicated (8 s at 66% amplitude using a Branson sonicator), clarified by a centrifugation step at 13,000 rpm for 15 min at 4 °C. Protein concentration was measured using a Pierce BCA Protein assay kit (Thermofisher Scientific, Geneva, Switzerland, #23225). A total of 10 µg of total proteins was loaded in each well of either 8% or 15% polyacrylamide SDS gels, and transferred onto nitrocellulose membrane for 2 h at 80 V at 4 °C. Nitrocellulose filters were incubated with the following primary antibodies overnight at 4 °C: anti-β-tubulin (Mouse, #32-250600, 1/2 500), anti-Claudin-2 (Mouse, #32-5600, 1/500), anti-Claudin-3 (Rabbit, #34-1700, 1/500), anti-Claudin-4 (Mouse, #32-9400, 1/500), and anti-Claudin-7 (Rabbit, #34-9100, 1/500). This was followed by HRP-conjugated secondary antibodies (Table S1) for 1 h at room temperature. Blots were developed using a WesternBright ECL kit (Advansta, San Jose, CA, USA, #K-12045-D50) and an Amersham ImageQuant 800 (Cytiva, Marlborough, MA, USA). Protein signals were quantified using Fiji/ImageJ. Relative signal intensities were expressed as ratios between the protein signals of interest and β-tubulin, used as a reference.

### 2.6. Plasmids

Constructs of pCDNA3.1(-)-eGFP-myc (S1166), pCDNA3.1(+)-myc-hZO-1-FL-HA (S1947), pCDNA3.1(−)-eGFP-cCGN-FL-myc (S1115), and pCDNA3.1(−)-eGFP-cCGNL1-FL-myc (S1148) have been described previously [24,30,31].

### 2.7. Immunofluoresence Anaylsis

For immunofluorescence (IF) analysis, MDCK clonal lines were co-cultured with WT cells on 12 mm Transwell filters seeded at a density of 1 × 10^5^ cells/well. For rescue IF experiments, double-KO cells were seeded and transfected 24 h later with 0.2 µg of plasmid DNA using jetOPTIMUS (Polyplus, VWR International AG, Nyon, Switzerland, #117-15). At confluence (72 h post-seeding and 48 h post-transfection), cells were washed with ice-cold PBS and fixed using cold methanol overnight at −20 °C, followed by a quick incubation in ice-cold acetone (1 min). Filters were excised manually using a razor blade, followed by rehydration in IMF buffer (0.1% TX-100, 0.15 M NaCl, 5 mM EDTA, 20 mM Hepes, pH = 7.5) and 2 washes in IMF buffer. Incubations with primary antibodies, anti-claudin-2 (Mouse, #32-5600, 1/50), anti-Claudin-1 (Rabbit, #51-9000, 1/50), anti-Claudin-3 (Rabbit, #34-1700, 1/100), anti-Claudin-4 (Mouse, #32-9400, 1/50), and anti-Claudin-7 (Rabbit, #34-9100, 1/100), anti-CGN (Rabbit, #C532, 1/5000), anti-GFP (Rabbit, #A11122, 1/200), anti-Myc (Rabbit, #06-549, 1/100), and anti-PLEKHA6 (Rat, #RtSZR127, 1/100) [32], were carried out at room temperature for 3 h (in a humidified chamber), followed by 3 washes in IMF buffer. Then, the filters were incubated with DAPI and secondary antibodies (1/300) for 2 h at room temperature, followed by 4 washes in IMF buffer. The filters were then placed on glass slides with the cells facing up and were mounted with Fluoromount-G. Slides were imaged on a Zeiss LSM800 confocal microscope using a Plan-Apochromat 63x/1.40 oil objective at a resolution of 1024 × 1024 px. Maximum intensity projections of z-stack images (typically 8-12 confocal planes, step size = 0.21 µm) were obtained. Images were extracted from .czi files using ImageJ and adjusted, cropped, and assembled using Affinity Designer. For the quantification of junctional immunofluorescent, the signal pixel intensity (mean gray value) for each channel was measured in the selected junctional area using the polyhedral tool of Fiji/ImageJ, and the averaged background signal of the image was subtracted. Relative fluorescence intensity (RFI) was expressed as a ratio between the signal of protein of interest (claudin-2) and an internal junctional reference (PLEKHA6) [32]. Ninety junctional segments per experimental condition were analyzed.

### 2.8. Quantification and Statistical Analysis

Data processing and analysis were carried out using GraphPad Prism8. All experiments were carried out in duplicate or triplicate. Data are shown as dot plots, histograms, or line graphs with means and standard deviations indicated. Statistical significance was determined by a one-way Anova. Normal distribution was verified with a Kolmogorov–Smirnov test, or by an unpaired Mann–Whitney test (ns: not significant, * *p* < 0.0332, ** *p* < 0.0021, *** *p* < 0.0002, **** *p* < 0.0001).

## 3. Results

### 3.1. MDCK Cells KO for Either CGN, CGNL1 or Both Show Slightly Increased Transepithelial Resistance but No Change in Permeability to Dextran

Clonal lines of MDCK cells KO for either CGN, CGNL1, or both (double-KO) were described previously and shown to lack expression of either CGN, CGNL1 or both by immunoblot (IB) and immunofluorescence microscopy (IF) [24,25]. CGN-KO and double-KO cells were identified by the lack of CGN immunofluorescent labeling, and CGNL1-KO cells were identified by exogenous expression of YFP, because of the low levels of endogenous CGNL1 expression in MDCK cells [24,33].

**Figure 1:**
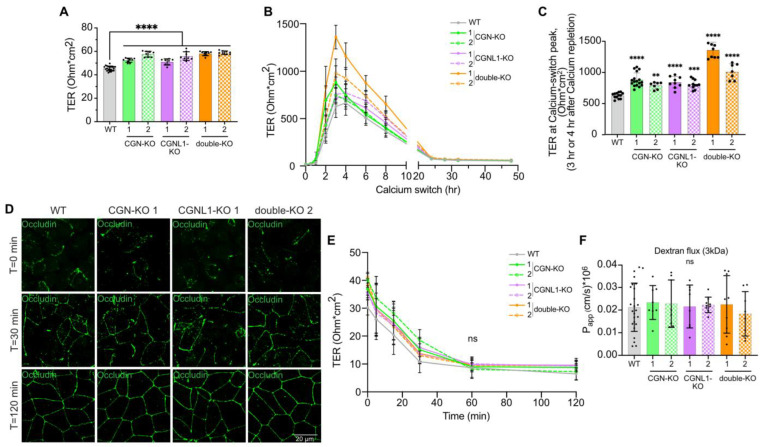
MDCK cells KO for either CGN or CGNL1 or both show slightly increased transepithelial resistance but no change in permeability to dextran. (**A**) TER measurement of confluent MDCK cells in the steady state. (**B**,**C**) TER profiles (T = 0 h to 48 h) (**B**) and peak values of TER (3 h for CGN-KO and double-KO, 4 h for WT, 3 h or 4 h for CGNL1-KO) (**C**) during the calcium switch of MDCK confluent cells. (**D**) Immunofluorescence microscopy analysis of occludin (green) after the calcium switch (T = 0 min to 120 min) in WT and KO for CGN, CGNL1 or both MDCK. Scale bar = 20 µm. Two biological replicates were carried out. (**E**) TER profiles during calcium depletion (T = 0 min to 120 min) of MDCK confluent cells. (**F**) Paracellular permeability to 3 kDa fluorescein-dextran measurement of confluent MDCK cells. (**A**–**C**,**E**,**F**) Genotypes and clone names are indicated on the *x*–axis (**A**,**C**,**F**) and upper right (**B**,**E**). Dots show technical replicates (*n* = 9–12) (**A**,**C**,**F**) or mean of technical replicates (**B**,**E**) and bars represent means ± SD for three biological replicates. Statistical significance of quantitative data was determined by a one-way Anova test (ns: not significant, ** *p* < 0.0021, *** *p* < 0.0002, **** *p* < 0.0001).

To assess whether the KO of either CGN or CGNL1 or both affects the paracellular barrier function, we first measured the transepithelial resistance (TER) of confluent monolayers in the steady state. The KO of either CGN or CGNL1 or both resulted in a small but statistically significant increase in the TER values compared to WT monolayers (increases of 15.4% and 26.9% for CGN-KO clone 1 and clone 2, respectively; 12.2% and 23.3% for CGNL1-KO clone 1 and clone 2, respectively; 27.8% and 29.4% for double-KO clone 1 and clone 2, respectively) (Figure 1A).

Next, we examined the dynamics of the formation and disruption of the paracellular barrier to ions by measuring the TER during junction assembly by the calcium switch protocol and junction disassembly by the calcium depletion protocol, respectively [34,35]. WT and KO clonal lines showed the characteristic profile of TER during junction assembly by the calcium switch, with a peak in the TER (Figure 1B). However, CGN-KO and double-KO reached the peak slightly earlier, at 3 h, compared to WT cells, which reached it 4 h after the beginning of the calcium switch, while CGNL1-KO cells reached a TER peak between 3 h and 4 h (Figure 1B). The KO lines, especially double-KO lines, showed a significantly higher value for the peak (increased by 39.9% and 25.5% for CGN-KO clone 1 and clone 2, respectively; 33.8% and 28.5% for CGNL1-KO clone 1 and clone 2, respectively; 116.4% and 60.8% for double-KO clone 1 and clone 2, respectively) (Figure 1B,C). To determine whether these differences correlated with a different dynamic of assembly of membrane protein components at the TJ, we carried out an immunofluorescence (IF) microscopy analysis of occludin. Occludin localization at the cell periphery was patchy at the start of the calcium switch (T = 0 min, Figure 1D) and was still discontinuous 30 min after the calcium switch in both WT and KO clones (Figure 1D). In contrast, 2 h after the switch, occludin labeling was detected in a continuous pattern along the junctions in both WT and KO lines (Figure 1D). Thus, the changes in the peak of the TER did not correlate with detectable changes in the dynamics of occludin assembly at junctions. Regarding the behavior of the paracellular barrier of KO cells following calcium depletion, the TER values of monolayers decreased rapidly, with a similar dynamic for WT and KO clones (Figure 1E).

Finally, we asked whether the loss of either CGN or CGNL1 or both affects the paracellular flux of 3 kDa fluorescein-dextran. In the steady state, the apparent permeability to dextran was not significantly different in WT cells and in clonal lines KO for either CGN or CGNL1 or both (Figure 1F).

In summary, the KO of either CGN or CGNL1 or both resulted in a minor but significant increase in the TER values both in the steady state and during the calcium switch, but had no detectable effect either on the dynamics of TJ barrier assembly or disassembly or on the permeability of 3 kDa fluorescein-dextran.

### 3.2. KO of Either CGN or CGNL1 or Both Results in Decreased Sodium Permeability of MDCK Monolayers

**Figure 2:**
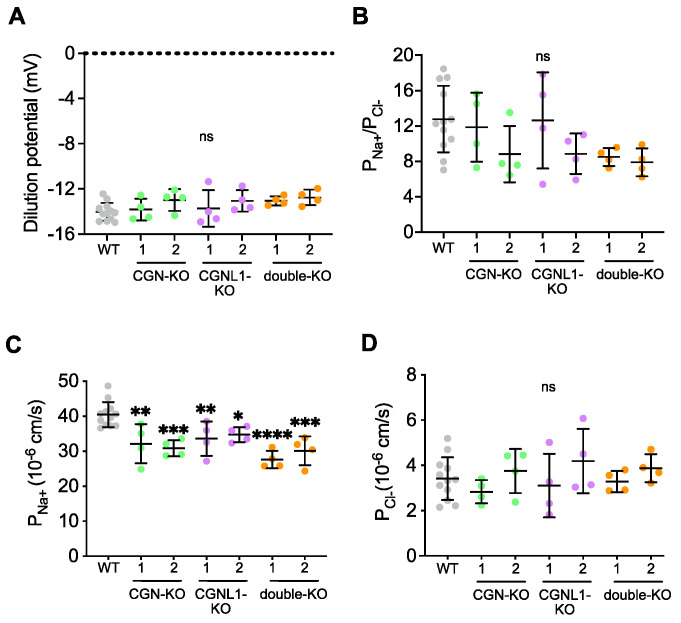
KO of either CGN or CGNL1 or both results in decreased sodium permeability of MDCK monolayers. (**A**–**D**) Dilution potential of NaCl (**A**), relative permeability ratio for Na^+^ over Cl^−^ (P_Na_^+^/P_Cl_^−^) (**B**), absolute permeabilities of Na^+^ (**C**) and Cl^−^ (**D**) of confluent MDCK cells. (**A**–**D**) Genotypes and clone names are indicated on the *x*–axis. Dots show four biological replicates and bars represent means ± SD. Statistical significance of quantitative data was determined by a one-way Anova test (ns: not significant, * *p* < 0.0332, ** *p* < 0.0021, *** *p* < 0.0002, **** *p* < 0.0001).

To further characterize the barrier properties of WT, CGN-KO, CGNL1 KO, or double-KO monolayers, we determined the absolute and relative ion permeabilities of sodium (Na^+^) and chloride (Cl^−^) using the method of dilution potentials [36]. The dilution potential and the paracellular permeability ratio for Na^+^ over Cl^−^ (P_Na+_/P_Cl_^−^) were not significantly decreased in MDCK monolayers KO for either CGN or CGNL1 or both when compared to WT (Figure 2A,B). However, the absolute permeability to Na^+^ was significantly decreased in CGN-KO (decreased by 20.25% for clone 1 and 23.75% for clone 2), in CGNL1-KO (decreased by 17.12% for clone 1 and 14.23% for clone 2), and in double-KO (decreased by 31.75% for clone 1 and 25.55% for clone 2) compared to WT (Figure 2C). In contrast, the KO of either CGN or CGNL1 or both did not affect the absolute permeability to Cl^−^ (Figure 2D).

These results suggest that KO of CGN, CGNL1, or both in MDCK cells modulates the paracellular barrier to ions by reducing Na^+^ permeability.

### 3.3. MDCK Cells KO for Either CGN or CGNL1 or Both Show Decreased Claudin-2 Protein Expression

**Figure 3:**
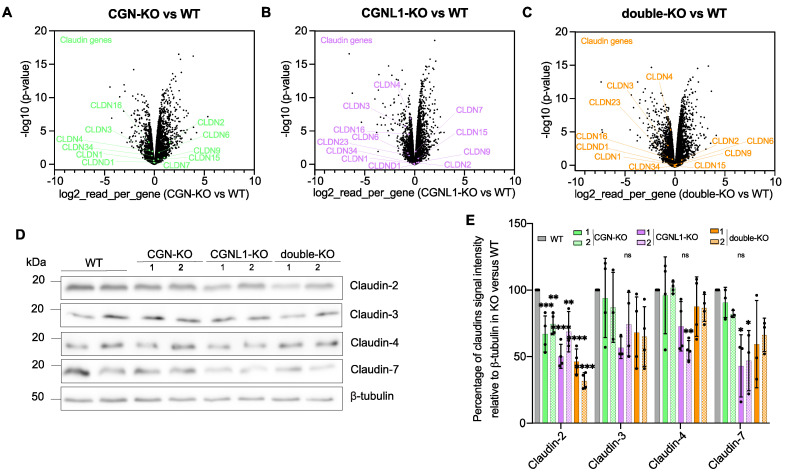
MDCK cells KO for either CGN or CGNL1 or both show decreased claudin-2 protein expression. (**A**–**C**) Volcano plots illustrating claudin mRNA levels in MDCK WT versus CGN-KO clone 1 (**A**), in WT versus CGNL1-KO clone 1 (**B**), and in WT versus double-KO clone 2 (**C**). Fold changes of reads per gene (log2, *x*–axis) and corresponding *p* values (-log10, y axis) are plotted. (**D**,**E**) Immunoblot analysis (**D**) and relative densitometric quantifications (**E**) of protein levels of claudin-2, -3, -4, and -7 in lysates of WT, and KO for CGN, CGNL1, or both MDCK cells. (**E**) Genotypes and clone names are indicated on the *x*–axis. β-tubulin was used as a loading control. Dots show three to four biological replicates and bars represent means ± SD. Statistical significance of quantitative data was determined by a one-way Anova (ns: not significant, * *p* < 0.0332, ** *p* < 0.0021, *** *p* < 0.0002, **** *p* < 0.0001).

The paracellular barrier to ions depends on claudins, which comprise pore-forming and barrier-forming claudins [7,37]. To determine if the changes in TER observed in MDCK cells KO for either CGN or CGNL1 or both correlate with altered expression of specific claudin isoforms, we measured the expression levels of mRNAs coding for claudins in KO versus WT cells in a micro-array analysis (Figure 3A–C). In agreement with previous observations, showing that levels of claudin-1 protein were not altered in MDCK cells KO for CGN, CGNL1, or both [25], the mRNA levels of claudin-1 (*CLDN-1)* were not impacted by the KO of either CGN or CGNL1 or both (Figure 3A–C). mRNA levels for most additional claudin isoforms that we analyzed (*CLDN-3, -4, -6, -7, -9, -15, -16, -23,* and *-34*) were not significantly altered in cells KO for either CGN or CGNL1 or both compared to WT (Figure 3A–C, Table 1, Table 2 and Table 3). However, claudin-2 (*CLDN-2*) mRNA levels were significantly increased (≈1.5-fold increase) in CGN-KO cells compared to WT (Figure 3A, Table 1), but not in CGNL1-KO and double-KO cells (Figure 3B,C, Table 2 and Table 3), suggesting that they may be due to clone-dependent variations.

Next, we examined protein expression of claudin-2, -3, -4, and -7 by immunoblot analysis of cell lysates, using β-tubulin as an internal standard (Figure 3D,E). Claudin-2 protein expression was slightly but significantly decreased in the KO of either CGN or CGNL1 or both compared to WT. For claudin-3, -4, and -7, no significant change in protein levels was observed in KO versus WT clonal cell lines, except for a slight and significant decrease in claudin-4 and claudin-7 protein expression in CGNL1-KO clone 1 and CGNL1-KO clones 1 and 2, respectively (Figure 3D,E).

Together, these results indicate that KO of either CGN or CGNL1 or both induces a decrease in claudin-2 protein expression, and the KO of CGN results in a small increase in claudin-2 mRNA levels.

### 3.4. MDCK Cells KO for CGN, CGNL1, or Both Show Decreased Claudin-2 Accumulation at Junctions

**Figure 4:**
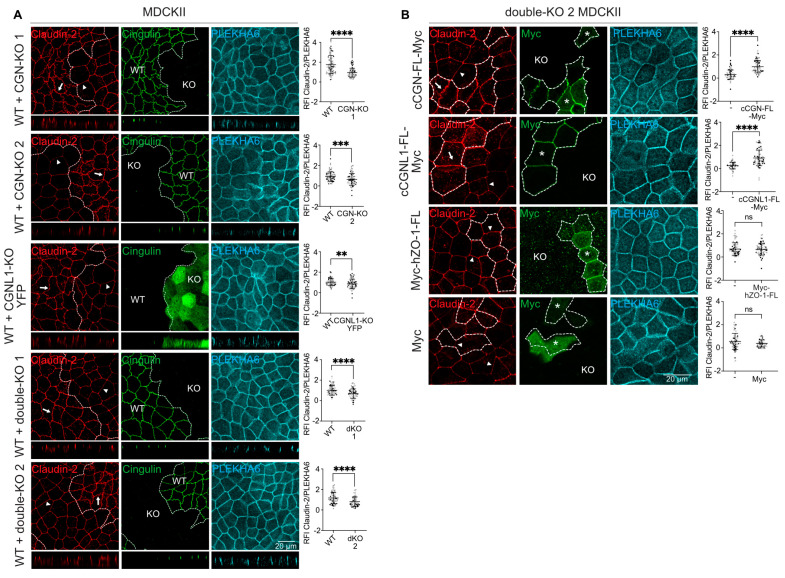
MDCK cells KO for CGN, CGNL1, or both show decreased claudin-2 accumulation at junctions. (**A**) Immunofluorescence microscopy analysis and relative fluorescence intensity (RFI) quantifications of endogenous claudin-2 (red) at junctions in mixed cultures of WT and KO either for CGN, CGNL1, or both; distinguished via CGN or GFP (green). Z sections taken at the horizontal middle positions are shown below XY images. (**B**) Immunofluorescence microscopy analysis and RFI quantifications of endogenous claudin-2 (red) at junctions in double-KO cells rescued with either full–length Myc–tagged canis CGN (cCGN–FL–Myc), full–length Myc–tagged canis CGNL1 (cCGNL1–FL–Myc), full–length Myc–tagged human ZO–1 (Myc–hZO–1–FL), or by Myc alone as a negative control; distinguished via Myc (green). Asterisks indicate rescued cells. (**A**,**B**) Arrow indicates normal junctional claudin-2 localization, arrowhead indicates junctional claudin-2 reduction. Quantification of RFI corresponds to the ratio between the junctional staining of claudin-2 and the junctional marker PLEKHA6 (cyan). Scale bar = 20 µm. Dots show technical replicates (*n* = 90 junctions) and bars represent means ± SD for three biological replicates. Statistical significance of quantitative data was determined by an unpaired Mann-Whitney test (ns: not significant, ** *p* < 0.0021, *** *p* < 0.0002, **** *p* < 0.0001).

Since claudin-2 promotes paracellular permeability to Na^+^ in MDCK cells [38,39,40,41] and we observed decreased Na^+^ permeability and claudin-2 protein expression in our KO lines, we quantitatively analyzed the junctional localization of claudin-2 by IF, using PLEKHA6 as an internal marker. The intensity of junctional claudin-2 labeling was slightly but significantly decreased in the CGN-KO, CGNL1-KO and double-KO clonal lines (arrowheads and histograms, Figure 4A) compared to WT (arrows and histograms, Figure 4A). When analyzed in Z sections, the relative distribution of claudin-2 labeling in the junctional versus lateral domains of the plasma membrane was similar in WT and KO cells (Z sections shown below XY images in Figure 4A), indicating that decreased junctional localization did not correlate with increased lateral accumulation.

To exclude the possibility that the decrease in junctional claudin-2 labeling in KO cells was due to clone-dependent variations, we rescued double-KO cells with either CGN or CGNL1 constructs. Expression of either myc-tagged full-length CGN (Figure 4B, top) or myc-tagged full-length CGNL1 (Figure 4B, top middle) in double-KO MDCK cells rescued normal claudin-2 junctional labeling, whereas expression of either myc-tagged full-length ZO-1 (Figure 4B, bottom middle) or myc alone (Figure 4B, bottom) did not.

We also analyzed the junctional localization of claudin-1, -3, -4, and -7. Their junctional localization was not affected by the KO of either CGN or CGNL1 or both when compared to WT cells (Figure S1A–D).

Together, IF and IB analyses indicated that CGN and CGNL1 are required for the efficient expression and junctional accumulation of endogenous claudin-2 at junctions in MDCK cells, whereas the junctional accumulation of claudin-1, -3, -4, and -7 is independent of CGN and CGNL1.

## 4. Discussion

**Figure 5:**
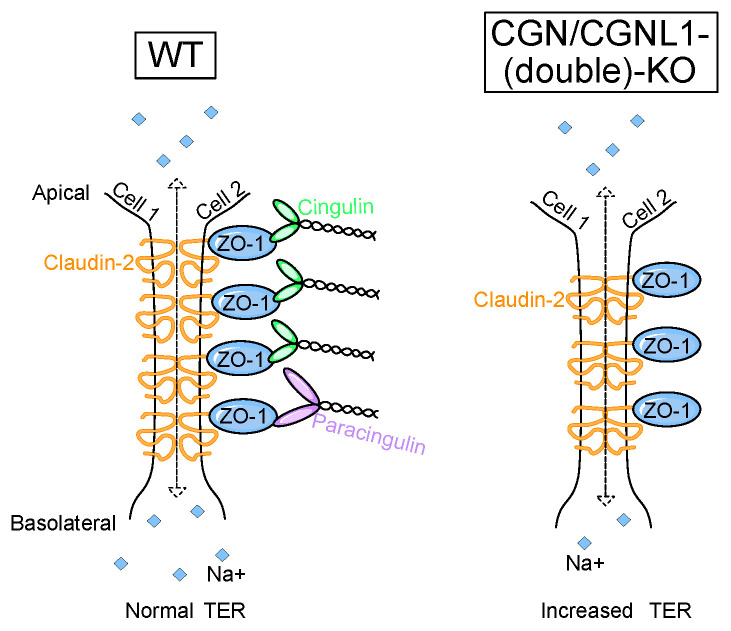
Simplified schematic model for the role of CGN and CGNL1 in barrier functions. (**Left panel**): organization of the TJ related to barrier function in WT MDCK. (**Right panel**): organization of the TJ related to barrier function in MDCK KO for CGN, CGNL1, or both.

Little is known about the role of junctional adaptor proteins in the regulation of the TJ barrier function in cells and tissues [2,5,10]. Here, we addressed this question for CGN and CGNL1, using a KO approach in MDCK cells, a well-established model system to study epithelial biology and TJ barrier functions [42]. This is the first time that a KO approach in an epithelial cultured cell model system has been used to investigate the role of CGN and CGNL1 in barrier functions. As summarized schematically in Figure 5, we found that the KO of either CGN or CGNL1 or both results in (1) a small but significant increase in transepithelial resistance in the steady state and during a calcium switch; (2) no significant effect on the dynamics of TJ assembly or disassembly; (3) no significant effect on permeability to 3 kDa fluorescein-dextran; (4) a slight but significant decrease in sodium permeability; (5) slightly increased mRNA levels and significantly decreased protein levels of claudin-2; and (6) a significant decrease in claudin-2 accumulation at junctions, which is rescued by expression of either CGN or CGNL1 but not ZO-1.

In previous studies, we have shown that neither shRNA-mediated depletion nor overexpression of CGN and CGNL1 affected the steady-state TER in MDCK epithelial cells. This is despite changes in the activation of RhoA and Rac1 GTPases, which in the case of CGNL1-KD cells correlated with a decrease in the TER peak during junction assembly by a calcium switch [20,21,43]. Moreover, consistent with current observations of MDCK KO cells, we previously did not detect any changes in the barrier permeability to large solutes in CGN-KO embryoid bodies [22] or in the electrical resistance of CGN-KO mouse intestinal mucosal preparations [23]. In this study using clonal KO cells, we observed a mild barrier phenotype, i.e., a small but significant increase in the TER both in the steady state and at the peak during TJ assembly induced by the calcium switch, with no change in the permeability to 3 kDa dextran. The TER phenotype of KO cells correlated with decreased permeability to Na^+^ and decreased expression and accumulation of claudin-2 at junctions. Since the loss of claudin-2 results in an increase in the TER and reduced permeability to Na^+^ [10,40,44,45], our results suggest that a higher TER and decreased Na^+^ permeability in MDCK cells KO for either CGN or CGNL1 or both are mechanistically linked to decreased junctional claudin-2. These results are unlikely to be due to clone-dependent variations, since each clonal line, including control WT cells, was generated independently and the reduced junctional claudin-2 was rescued by re-expression of either CGN or CGNL1, but not by ZO-1.

The mechanism through which the KO of either CGN or CGNL1 or both leads to reduced claudin-2 at junctions is unclear. Since ZO-1 is a scaffold for claudins [15], one possibility to account for decreased claudin-2 is a decrease in junctional ZO-1. However, only the KO of CGN, but not that of CGNL1, resulted in a decreased accumulation of ZO-1 at TJs [25], whereas decreased claudin-2 was observed for both CGN-KO and CGNL1-KO clones. Moreover, ZO-1 overexpression did not rescue the normal accumulation of claudin-2. Thus, it is unlikely that either CGN or CGNL1 promotes the junctional accumulation of claudin-2 by regulating ZO-1. No detectable change in TJ strand organization was observed in CGN-KO embryoid bodies [22], suggesting that changes in claudin-2 levels are not correlated with altered TJ strand morphology, which could affect claudin-2 integration into strands. Alternative hypotheses, including a different association of claudin-2 with the actomyosin cytoskeleton and different rates of degradation and endocytosis, should be investigated in future studies. Interestingly, we have previously shown that depletion of either CGN or CGNL1 by sh-RNA resulted in increased claudin-2 expression both at the mRNA and protein levels [20,21]. In addition, KO of CGN in mice results in increased claudin-2 protein levels in the kidneys and intestinal epithelia [23]. Our results therefore indicate that KD and KO models in MDCK cells and KO models in MDCK versus mice exhibit opposite phenotypes with respect to claudin-2 expression and junctional accumulation. Moreover, while CGN-KD and CGNL1-KD MDCK cells showed altered activation of RhoA and Rac1 activities, which appeared to be mechanistically involved in increasing claudin-2 expression [20], no changes in RhoA and Rac1 activation or downstream phenotypes have been detected in MDCK cells KO for either CGN or CGNL1 or both [24] or in mouse tissues [23]. Thus, expression and junctional accumulation of claudin-2 appear to be potentially regulated by CGN and CGNL1 through multiple pathways, which are differentially affected by the type of depletion approach and the model system.

Overall, the results reported here confirm our previous conclusion [23] that CGN is dispensable in the epithelial barrier function and also indicate that CGNL1 is not redundantly required with CGN for epithelial barrier function. Our results also indicate that reduced TJ membrane tortuosity, which is detected in MDCK cells KO for CGN and CGNL1 together with decreased apical membrane stiffness [24], does not correlate with major changes in the TJ barrier function. However, the cellular context, the molecular environment, and specific physio-pathological stimuli may be critical in highlighting the potential role of CGN and CGNL1 in TJ barrier functions. For example, CGN-KO and CGN-KD endothelial cells and tissues show altered barrier function in vitro and in vivo, correlating with increased GEF-H1-dependent RhoA activity upon depletion of CGN [17,18,19]. This suggests that epithelial cells may contain redundant molecular complexes and mechanisms and that endothelial cells are lacking to compensate for the loss of either CGN or CGNL1 or both. Importantly, both CGN-KO and CGNL1-KO mice are viable and apparently healthy [23,46], suggesting that any change in endothelial TJ permeability resulting from a loss of either CGN or CGNL1 does not impair growth and survival of mice under normal conditions. However, because of their implication in the regulation of Rho family GTPases and multiple cytoskeletal interactions, CGN and CGNL1 may still play a role in epithelial and endothelial barrier function under conditions where the barrier is challenged by pathogens, cytokines, or other physiological and pathological stimuli. Additional studies will be required to address these hypotheses in cultured cell model systems in vitro and in tissues in vivo.

## 5. Conclusions

The present study supports our previous results indicating that CGN and CGNL1 are not required to maintain TJ barrier functions in epithelial monolayers. Clonal lines of MDCK cells KO for CGN, CGNL1, or both displayed a small increase in TER, which was correlated with a decreased sodium permeability and decreased claudin-2 expression and accumulation at junctions.

## Figures and Tables

**Table 1:** Claudin isoform mRNA expression in WT versus CGN-KO MDCK cells.

WT vs. CGN-KO 1			
Gene	Protein	Fold Change of Reads per Gene (log2)	*p*-Values (−log10)
*CLDN1*	Claudin-1	−0.063447	0.591236046
*CLDN2*	Claudin-2	1.53523337	3.28460007
*CLDN3*	Claudin-3	−0.499567	2.142506518
*CLDN4*	Claudin-4	−0.1789856	1.934767597
*CLDN6*	Claudin-6	0.46861894	1.772412443
*CLDN7*	Claudin-7	0.02805951	0.249124176
*CLDN9*	Claudin-9	0.99625261	1.018853025
*CLDN15*	Claudin-15	0.38513126	0.797467008
*CLDN16*	Claudin-16	−0.5573263	3.327300283
*CLDN23*	Claudin-23	−0.6045539	1.163347307
*CLDN34*	Claudin-34	−0.253871	1.311607839

**Table 2:** Claudin isoform mRNA expression in WT versus CGNL1-KO MDCK cells.

WT vs. CGNL1-KO 1			
Gene	Protein	Fold Change of Reads per Gene (log2)	*p*-Values (−log10)
*CLDN1*	Claudin-1	0.01875057	0.135071785
*CLDN2*	Claudin-2	0.21523078	0.232246767
*CLDN3*	Claudin-3	−0.9139707	4.300967584
*CLDN4*	Claudin-4	−0.4097344	5.170136642
*CLDN6*	Claudin-6	−0.4098655	1.30666783
*CLDN7*	Claudin-7	0.11067803	1.478666107
*CLDN9*	Claudin-9	0.39999074	0.272456048
*CLDN15*	Claudin-15	0.35576133	0.696931579
*CLDN16*	Claudin-16	−0.5752402	3.413889287
*CLDN23*	Claudin-23	−0.5657903	1.031935294
*CLDN34*	Claudin-34	−0.0403864	0.13166079

**Table 3:** Values of claudin mRNA levels in WT versus double-KO MDCK cells.

WT vs. Double-KO 2			
Gene	Protein	Fold Change of Reads per Gene (log2)	*p*-Values (−log10)
*CLDN1*	Claudin-1	0.0446277	0.377016368
*CLDN2*	Claudin-2	0.67800971	1.059246572
*CLDN3*	Claudin-3	−0.7014976	3.236609447
*CLDN4*	Claudin-4	−0.4432871	5.592565053
*CLDN6*	Claudin-6	0.16348518	0.420113214
*CLDN7*	Claudin-7	0.03413621	0.315691802
*CLDN9*	Claudin-9	0.63773487	0.518749023
*CLDN15*	Claudin-15	0.06764114	0.093204682
*CLDN16*	Claudin-16	−0.247682	1.166960751
*CLDN23*	Claudin-23	−0.7573273	1.517074677
*CLDN34*	Claudin-34	−0.0426028	0.14099429

## Data Availability

Datasets generated during the study will be archived upon acceptance of the manuscript.

## Supplementary Materials

The following supporting information can be downloaded at: https://www.mdpi.com/article/10.3390/cells12152004/s1, Figure S1: Junctional localization of claudin-1, -3, -4, and -7 is not affected by the KO of either CGN or CGNL1 or both in MDCK cells. Table S1: Resource Table.

## Abbreviations

TJ, tight junctions; Na+, sodium; CGN, cingulin; CGNL1, paracingulin; MLC, myosin light chain; TER, transepithelial resistance; KD, knock down; KO, knock out; Cl^−^, chloride; CLDN, claudin; IF, immunofluorescence; NMIIB, non-muscle myosin IIB; Madin–Darby Canine Kidney-II, MDCK; DMEM, Dulbecco’s Modified Eagle’s; FBS, Fetal Bovine Serum; NEAA, non-essential amino-acids; P/S, penicillin and streptomycin; Perm_app_, apparent permeability; PBS, phosphate-buffered saline; DPBS, Dulbecco’s phosphate-buffered saline; S-MEM, suspension-minimum essential medium; EGTA, ethylene glycol-bis(β-aminoethyl ether)-N,N,N’,N’’-tetraacetic acid, NaCl, sodium chloride; NaHCO_3_, sodium bicarbonate; KCl, potassium chloride; CaCl_2_, calcium chloride; MgCl_2_, magnesium chloride; O_2_, oxygen; CO_2_, carbon dioxide; GLM, general linear model; FC, fold change; FDR, false discovery rate; RIPA, radioimmunoprecipitation assay buffer; EDTA, ethylenediaminetetraacetic acid; SDS, sodium dodecyl sulfate; FL, full length; DAPI, 4′,6-diamidino-2-phenylindole; GFP, green fluorescent protein; RT, room temperature; PLEKHA6, Pleckstrin Homology Domain-containing, family A member 6; kDa, kilodaltons; ECL, enhanced chemiluminescence; SD, standard deviation; Px, pixels; RFI, relative fluorescence intensity; ns, not significant; WT, wild type; ZO-1, zonula occludens-1; IB, immunoblot.
