# Supplementary material for: Knock Out of CGN and CGNL1 in MDCK Cells Affects Claudin-2 but Has a Minor Impact on Tight Junction Barrier Function
Source: Cells. 2023 Aug 5;12(15):2004. doi: 10.3390/cells12152004 (PMC10417749; doi:10.3390/cells12152004)
Supplement: Supplementary file 1 [file cells-12-02004-s001.zip › cells-2496141-supplementary.pdf]

SUPPLEMENTARY MATERIAL

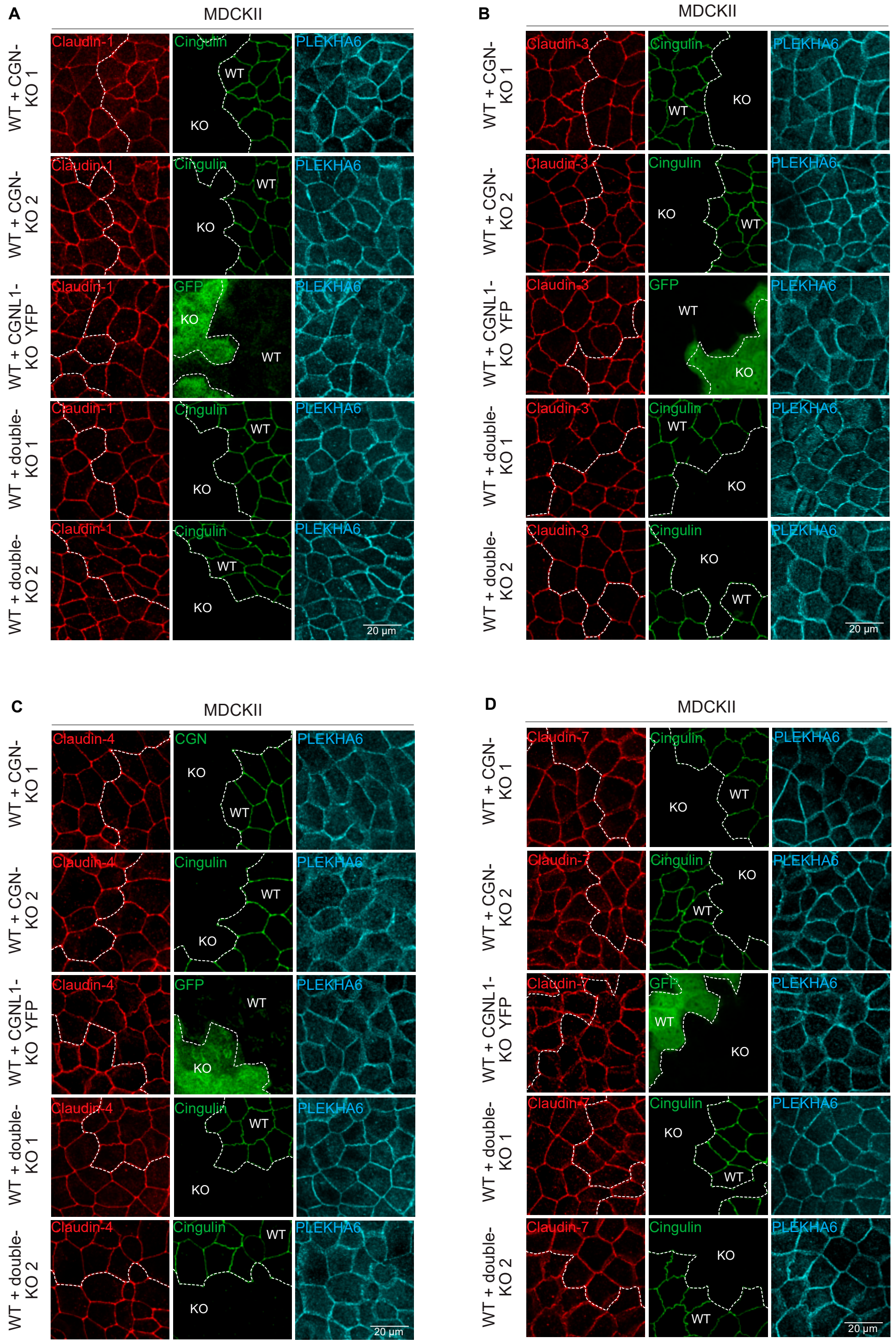

Figure S1. The junctional localization of claudins -1, -3, -4, -7 is not affected by the KO of either CGN, CGNL1 or both in MDCK cells. (**A-D**). IF microscopy analysis of endogenous claudin-1 (**A**), claudin-3 (**B**), claudin-4 (**C**) and claudin-7 (**D**) (red) at junctions of mixed cultures of WT cells, and cells KO either for CGN, CGNL1 or both. KO cells were identified either by lack of CGN labeling or by cytoplasmic GFP (green). PLEKHA6 was used as junctional marker reference. Dotted lines mark the border between WT and KO cells. Scale bar = 20 µm.

Table S1. Resources table.

Reagents and resources	Source	Identifier
**Antibodies**		
Rabbit polyclonal anti-cingulin (IB, IF)	Citilab	C532
Rabbit polyclonal anti-paracingulin (IB)	Citilab	20893
Mouse monoclonal anti-b-tubulin (IB)	Thermo Fisher Scientific	Cat# 32-2600<br>RRID: AB_2533072
Mouse monoclonal anti-Claudin-2 (IB, IF)	Thermo Fisher Scientific	Cat# 32-5600<br>RRID: AB_2533085
Rabbit polyclonal anti-Claudin-3 (IB)	Thermo Fisher Scientific	Cat# 34-1700<br>RRID: AB_2533158
Mouse monoclonal anti-Claudin-4 (IB)	Thermo Fisher Scientific	Cat# 32-9400<br>RRID: AB_2533096
Rabbit polyclonal anti-Claudin-7 (IB)	Thermo Fisher Scientific	Cat# 34-9100<br>RRID: AB_2533190
Rabbit polyclonal anti-GFP (IF)	Thermo Fisher Scientific	Cat# A-11122<br>RRID: AB_221569
Rabbit polyclonal anti-myc (IF)	Sigma-Aldrich/Merck	Cat# 06-549<br>RRID:
Rat polyclonal anti-PLEKHA6 (IF)	[1]	RtSZR127
Cy3-AffiniPure Donkey anti-Mouse IgG	Jackson Laboratory	Cat# 715-165-151<br>RRID: AB_2315777
Cy3-AffiniPure Donkey anti-Rat IgG	Jackson Laboratory	Cat# 712-166-150<br>RRID: AB_2340668
Alexa Fluor 488-AffiniPure Donkey anti-Rabbit IgG	Jackson Laboratory	Cat# 711-545-152<br>RRID: AB_2313584
Cy5-AffiniPure Donkey anti-Rat IgG	Jackson Laboratory	Cat# 712-175-153<br>RRID: AB_2340672
Cy5-AffiniPure Donkey anti-Mouse IgG	Jackson Laboratory	Cat# 715-175-150<br>RRID: AB_2340819
Anti-mouse IgG (H+L), HRP Conjugate	Promega	Cat# W4021<br>RRID: AB_430834
Anti-rabbit IgG (H+L), HRP Conjugate	Promega	Cat# W4011<br>RRID: AB_430833
**Plasmids**		
pCDNA3.1(-)-eGFP-myc-his	[2]	S1166
pCDNA3.1(+)-myc-hZO-1-FL-HA	[3]	S1947
pCDNA3.1(-)-eGFP-cCGN-FL-myc	[4]	S1115
pCDNA3.1(-)-eGFP-cCGNL1-FL-myc	[4]	S1148
**Chemicals, Reagents, Critical commercial assays**		
Pierce Protease Inhibitor Tablet, EDTA-free	Thermo Scientific	Cat# A32965
jetOPTIMUS	Polyplus	Cat# 117-15
Hanks buffer	Gibco	Cat# 14025-050
3 kDa fluorescein-dextran	Invitrogen	Cat# D3305
Ca^{2+}-free DPBS	Gibco	Cat# 14190-094
S-MEM (Gibco, #11380-037)	Gibco	Cat# 11380-037

Amiloride	Sigma-Aldrich/Merck	Cat# A-7410
DIDS	Sigma-Aldrich/Merck	Cat#D3514
NucleoSpin® RNA kit	Macherey-Nagel	Cat# 740955.50
Piece BCA Protein assay kit	Thermo Scientific	Cat# 23225
WesternBright ECL kit	Advansta	Cat# K-12045-D50
**Experimental models: Cell lines**		
MDCK (Madin-Darby Canine Kidney) Tet-Off	A Fanning, University of North Carolina	Clontech
MDCK (Madin-Darby Canine Kidney) Tet-Off CGN-KO	[5]	N/A
MDCK (Madin-Darby Canine Kidney) Tet-Off CGNL1-KO-YFP-myc	[5]	N/A
MDCK (Madin-Darby Canine Kidney) Tet-Off CGN/CGNL1-double-KO	[5]	N/A
**Software and algorithms**		
Image J	N/A	Imagej.nih.gov/ij/ RRID: SCR_003070
Affinity Designer	N/A	https://affinity.serif.com/ RRID: SCR_016952
Prism GraphPad	N/A	https://www.graphpad.com/scientific-software/prism/ RRID: SCR_002798

1. Sluysmans, S.; Mean, I.; Xiao, T.; Boukhatemi, A.; Ferreira, F.; Jond, L.; Mutero, A.; Chang, C.J.; Citi, S. PLEKHA5, PLEKHA6 and PLEKHA7 bind to PDZD11 to target the Menkes ATPase ATP7A to the cell periphery and regulate copper homeostasis. *Mol Biol Cell* **2021**, *32*, 1-20, doi:10.1091/mbc.E21-07-0355.
2. Guerrera, D.; Shah, J.; Vasileva, E.; Sluysmans, S.; Mean, I.; Jond, L.; Poser, I.; Mann, M.; Hyman, A.A.; Citi, S. PLEKHA7 Recruits PDZD11 to Adherens Junctions to Stabilize Nectins. *J Biol Chem* **2016**, *291*, 11016-11029, doi:10.1074/jbc.M115.712935.
3. Spadaro, D.; Le, S.; Laroche, T.; Mean, I.; Jond, L.; Yan, J.; Citi, S. Tension-Dependent Stretching Activates ZO-1 to Control the Junctional Localization of Its Interactors. *Curr Biol* **2017**, *27*, 3783-3795 e3788, doi:10.1016/j.cub.2017.11.014.
4. Rouaud, F.; Huang, W.; Flinois, A.; Jain, K.; Vasileva, E.; Di Mattia, T.; Mauperin, M.; Parry, D.A.D.; Dugina, V.; Chaponnier, C.; et al. Cingulin and paracingulin tether myosins-2 to junctions to mechanoregulate the plasma membrane. *J. Cell Biol.* **2023**, *322*, e202208065.
5. Vasileva, E.; Spadaro, D.; Rouaud, F.; King, J.M.; Flinois, A.; Shah, J.; Sluysmans, S.; Mean, I.; Jond, L.; Turner, J.R.; et al. Cingulin binds to the ZU5 domain of scaffolding protein ZO-1 to promote its extended conformation, stabilization, and tight junction accumulation. *J Biol Chem* **2022**, *298*, 101797, doi:10.1016/j.jbc.2022.101797.
